# PAX-Interacting Protein 1 (PTIP) Promotes Apoptosis

**DOI:** 10.33696/signaling.6.142

**Published:** 2025

**Authors:** Ching-Jung Huang, Hyein Cho, Chuan Li, Kangsan Kim, Danyang Yu, Daechan Park, Y. Jessie Zhang, Haley O. Tucker

**Affiliations:** 1 Department of Biology, Arts and Sciences, New York University in Shanghai, Shanghai 200122, China; 2 Department of Molecular Science and Technology, Advanced College of Bio-Convergence Engineering, Ajou University 206 Worldcup-ro, Yeongtong-gu, Suwon 16499, South Korea; 3 Molecular Biosciences, University of Texas at Austin, 1 University Station A5000, Austin, TX 78712, USA

**Keywords:** Breast cancer C-Terminal, Class switch recombination, DNA, Immunoglobulin

## Abstract

PAX-interacting protein 1 (PTIP/PAXIP1) was discovered and initially characterized over three decades ago as a 1,056 amino acid-containing protein with six tandem BReast cancer C-Terminal (BRCT) repeats. PTIP functions broadly to catalyze histone methylation in DNA damage repair and within the hematopoietic lineage, to promote immunoglobulin variable region (variable, diversity, joining [VDJ]) and class switch recombination (CSR). In this report, we show that a fraction of PTIP is actively transported from the nucleus to mitochondria resulting in their aggregation, release of cytochrome c into the cytoplasm and cellular apoptosis. Deletion of an N-terminal glutamine-rich region (QR), mutation of a conserved threonine within BRCT3 and truncation of the C-terminal BRCT5 domain each significantly reduced apoptosis as well as its previously documented G_2_/M cell cycle function. This is the first report to identify a mitochondrial-based apoptotic mechanism employed by the PTIP transcription factor.

## Introduction

**Figure 1.**
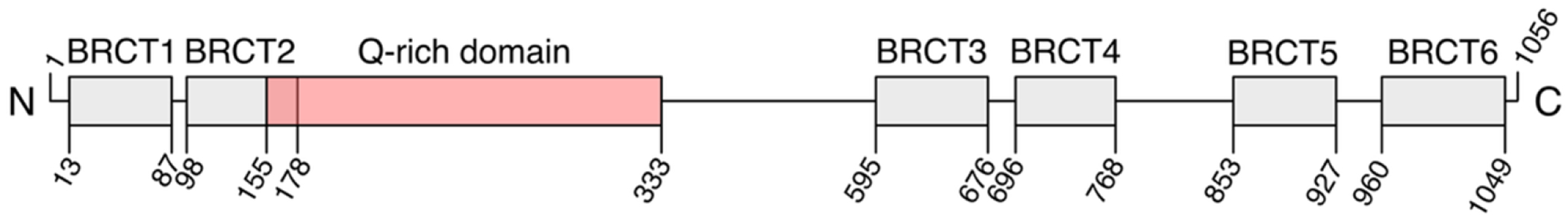
Motifs and domains in PTIP. The six black boxes represent BRCT domains, the pink box represents the QR domain. The corresponding amino acids are shown at ends of each domain. **Abbreviations:** BRCT: BReast Cancer C-Terminal; PTIP: PAX-Interacting Protein 1; QR: Glutamine-rich.

PAX Interacting Protein 1 (PTIP/PAXIP1) was first characterized in the mouse (m) as a 1,056 amino acid protein [1] with its most notable feature being a tandem array of 6 BReast cancer C-Terminal (BRCT) domains; two at its amino terminus and the other four within the carboxyl terminal region (Figure 1). BRCTs are found across evolution, from archaea to eukaryote and typically contain two 90–100 residue stretches, often in tandem and near the C-termini as reviewed recently by Pena-Guerrero *et al.* [2]. Most often BRCT-containing proteins, such as PTIP, which carry 6 C-terminal BRCTs, are involved in DNA damage response and in transcriptional regulation via complex formation with histone methyltransferases. BRCT proteins function during early development in DNA damage response and are required for cell survival following ionizing radiation. BRCT-containing proteins, at least *in vitro*, are involved in homologous recombination-mediated repair of double-strand breaks (DSBs) as recently reviewed [3].

The first molecular evidence that suggested a methylation-independent role for PTIP in transcription was revealed in the immune system. Callen *et al.* [4] found that PTIP regulated cleavage and repair during variable (V), diversity (D) and joining (J) (VDJ recombination) segments of the T cell receptor CD4+CD8+ double positive (DP) immature thymocytes. Shortly thereafter, PTIP was shown to regulate class switch recombination (CSR), an essential mechanism to diversify the adaptive immune response as well as to maintain B lymphocyte genome stability [5]. A number of studies employing conditional B cell-specific, Cre deletion [6–11], Daniel and Nussenzweig [11] concluded that the PTIP requirement in VDJ and CSR act both independent and dependent of its role in histone methylation.

Apoptosis, a non-lytic cell-death program, was initially defined by morphological features, which include nuclear condensation, cytoplasmic vacuolation, condensation, and plasma membrane blebbing as recently reviewed by Newton *et al.* [12]. Briefly, BCL2 (B-cell leukemia/lymphoma 2) prosurvival members block BAX (Bcl-2–associated X protein) and BAK (BCL2 Antagonist/Killer 1) prior to an apoptotic signal. On receipt of an apoptotic signal, BH3-containing factors bind to BAX and BAK forming oligomers, resulting in mitochondrial outer membrane permeabilization (MOMP). This releases cytochrome c into the cytoplasm where it activates the initiator Caspase-9. Caspase-3 and −7, which are proteolytically activated by caspase-9, execute the cleavage events that disassemble the apoptotic cell.

Heretofore, PTIP has not been shown to catalyze apoptotic activity. In this report we demonstrate that PTIP is transported from the nucleus via a classical nuclear localization mechanism to mitochondria resulting in their aggregation and release of cytochrome c into the cytoplasm. Mutation or deletion of BRCT3 and BRCT5 as well as deletion of a lengthy N-terminal-proximal polyglutamine repeat (QR) significantly reduced PTIP-mediated apoptosis as well as its previously documented G2/S cell cycle arrest capacity. We end with speculations regarding the mechanism by which PTIP is transferred to mitochondria and how large cytoplasmic aggregates, generated during this process are formed.

## Results

### PTIP/PAXIP1 and its domains

The domains relevant to the present study, further characterized from a generous gift from Dr. Greg Dressler [1], are illustrated in Figure 1. The complete PTIP sequence is available from GenBank (accession no. AF104261). Its predicted open reading frames (ORF) of 1,056 amino acids predict a protein of ~100kB and a transcript of ~3.4 kB. Notable features include an acidic region (36%), a putative nuclear localization signal (amino acids 834-847) and an extended region highly enriched in glutamine (QR domain; residues 178-333). This region is >63% glutamine with several long polyglutamine repeats of uninterrupted lengths varying from 6 to 13. PTIP contains 6 BRCT domains [1,13].

### PTIP is toxic when overexpressed

In attempts to characterize PTIP function *in vivo*, we consistently failed to establish stably transformed cell lines in which PTIP protein was constitutively expressed under the control of the CMV (Cytomegalovirus) promoter. This suggested that its over-expression is toxic to cells. The recipients included Bcl-1 leukemic B cells as well as NIH-3T3, Hela and 293 cells. These failures were confirmed by our inability to establish stably cell lines in which expression of GFP (Green fluorescent protein) or HA-tagged-PTIP was induced by addition of tetracycline to the culture medium. However, we succeeded by employing an N-terminal fusion of PTIP with GFP in which expression was under constitutive CMV promoter control (details of these constructions are provided in the Materials and Methods).

**Figure 2.**
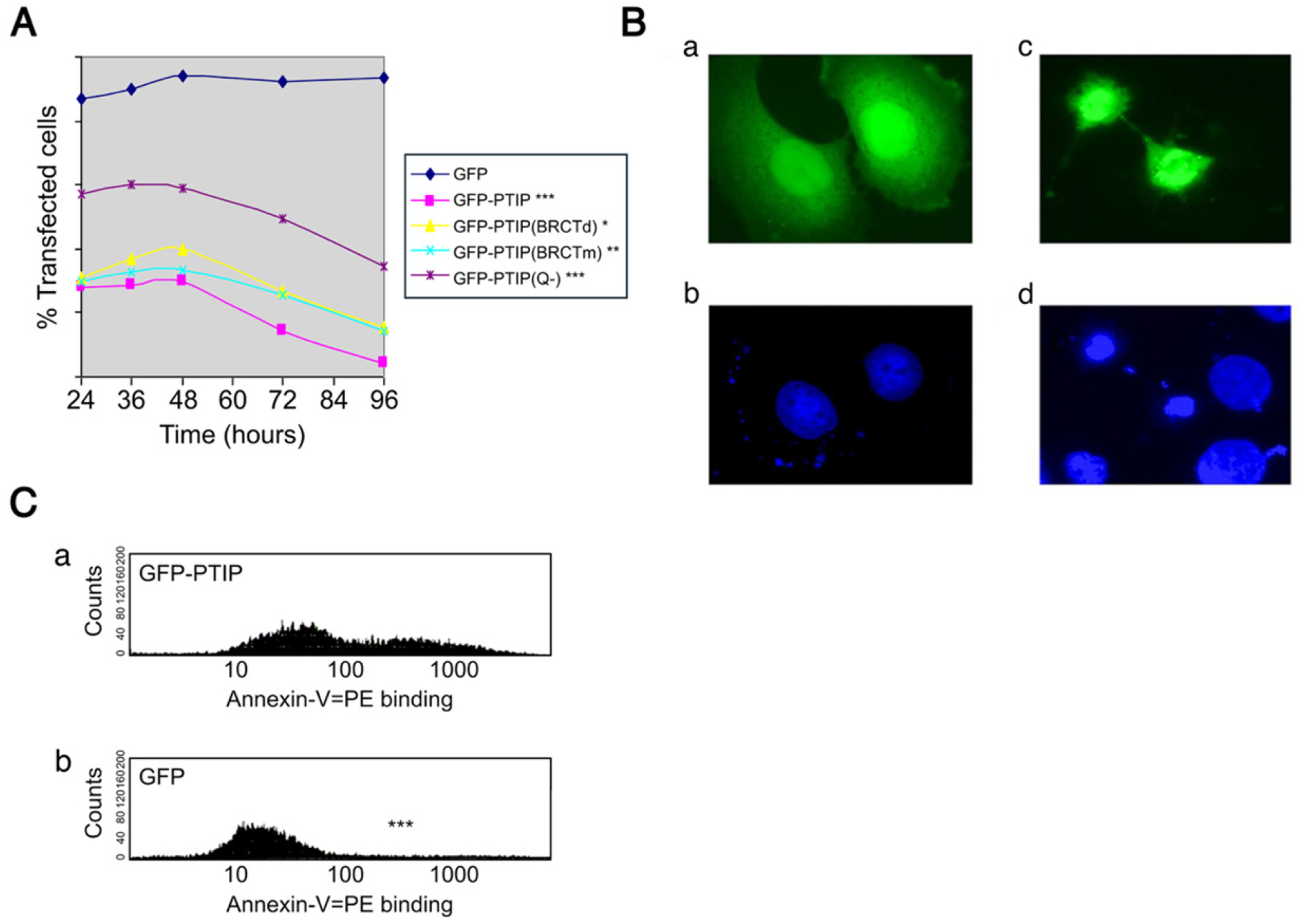
PTIP overexpressing cells die by apoptosis. All experimental values were determined as the average of 3 independent experiments with significance values indicated as p≤0.05*; p≤0.01**; p≤0.001***. (**A**) Killing curves of GFP-PTIP and its mutants. NIH-3T3 cells were transfected with 1.0 μg pEGFP, 1.0 μg pEGFP-PTIP, 1.0 μg pEGFP-PTIP(BRCTd) or 1.0 μg of pEGFP-PTIP(QR-). The percentage of transfected cells was determined by FACS at the indicated time points post transfection. Percentages of live cells were assessed. Data shown are averages of 3 independent experiments. (**B**) Morphological changes of GFP-PTIP transfected apoptotic Hela cells. a) Normal Hela cells transfected by GFP as a control; b) DAPI staining of A; c) Two apoptotic Hela cells transfected by GFP-PTIP; d) DAPI staining of C. Cells were observed 72 hours following transfection. (**C**) Annexin-V binding activity of GFP-PTIP. ~72 hr following transfection with 1μg of pEGFP (lower panel) or pEGFP-PTIP (upper panel), cells were stained by Annexin-V-PE (BD Pharmingen). The transfected cells were gated and analyzed by flow cytometry as described in Materials and Methods. **Abbreviations:** BRCT: BReast Cancer C-Terminal; DAPI: 4’,6-diamidino-2-phenylindole; FACS: Fluorescence-Activated Cell Sorting; GFP: Green Fluorescent Protein; PTIP: PAX-Interacting Protein 1; QR: Glutamine-rich.

As an alternative approach, NIH-3T3 cells were transiently transfected with pEGFP-PTIP, and the percentage of viable transfected cells was determined by flow cytometry at different time post transfection. As shown in Figure 2A, the percentage of viable pEGFP-PTIP transfectants decreased ~7-fold from 24 hrs to 96 hrs, while no significant change was observed in GFP-vector-only controls. Under fluorescence microscopy, we observed that at 72 or 96 hrs after post-transfection, most remaining PTIP transfectants showed classic morphological changes of apoptotic cells. That GFP vector-only transfectants remained viable allowed us to rule out the possibility that the decrease was caused by outgrowth of untransfected cells.

To rule out the possibility that the toxicity was a vector artifact, we observed similar, albeit less dramatic, results employing an HA-tagged PTIP or additional transfection recipients, including Bcl-1 mouse mature B cells, J558 mouse plasmacytomas, Hela, 293, COS-7 (CV-1 in Origin with *SV40* genes) or CHO (Chinese hamster ovary) cells (data now shown). We did note that 293 and COS-7 cells were more resistant to PTIP toxicity, which in retrospect may have resulted from the high expression level of anti-apoptotic Bcl-2 in this cell line (Dr. Xiaodong Wang, personal communication).

### PTIP overexpressing cells die by apoptosis

To determine whether the toxic effect was caused by apoptosis or necrosis, several criteria of apoptosis were tested. We first looked at the morphological changes of the PTIP transfected cells. Hela cells or NIH-3T3 cells were transfected by pEGFP-PTIP and analyzed 72 or 96 hours (hr) later. As shown in Figure 2B, their morphology was typical of apoptosis, including rounded cell shape, cell shrinkage, loss of contact with adjacent cells, as well as condensed and fragmented nuclear DNA. We observed that 72 hr post transfection, while few GFP-transfected CHO cells showed modest Annexin-V binding, over 40% of GFP-PTIP transfectants were Annexin-V positive (Figure 2C). As further support of apoptosis, GFP-PTIP cells at 72 hr post-transfection displayed sub-diploid DNA as judged by propidium iodine (PI) staining of >27%, as judged by flow cytometry (Figure 2C), demonstrating that overexpression of PTIP caused the loss of DNA content.

### PTIP-induced apoptosis can be inhibited by caspase inhibitors and by expression of Bcl-2

**Figure 3.**
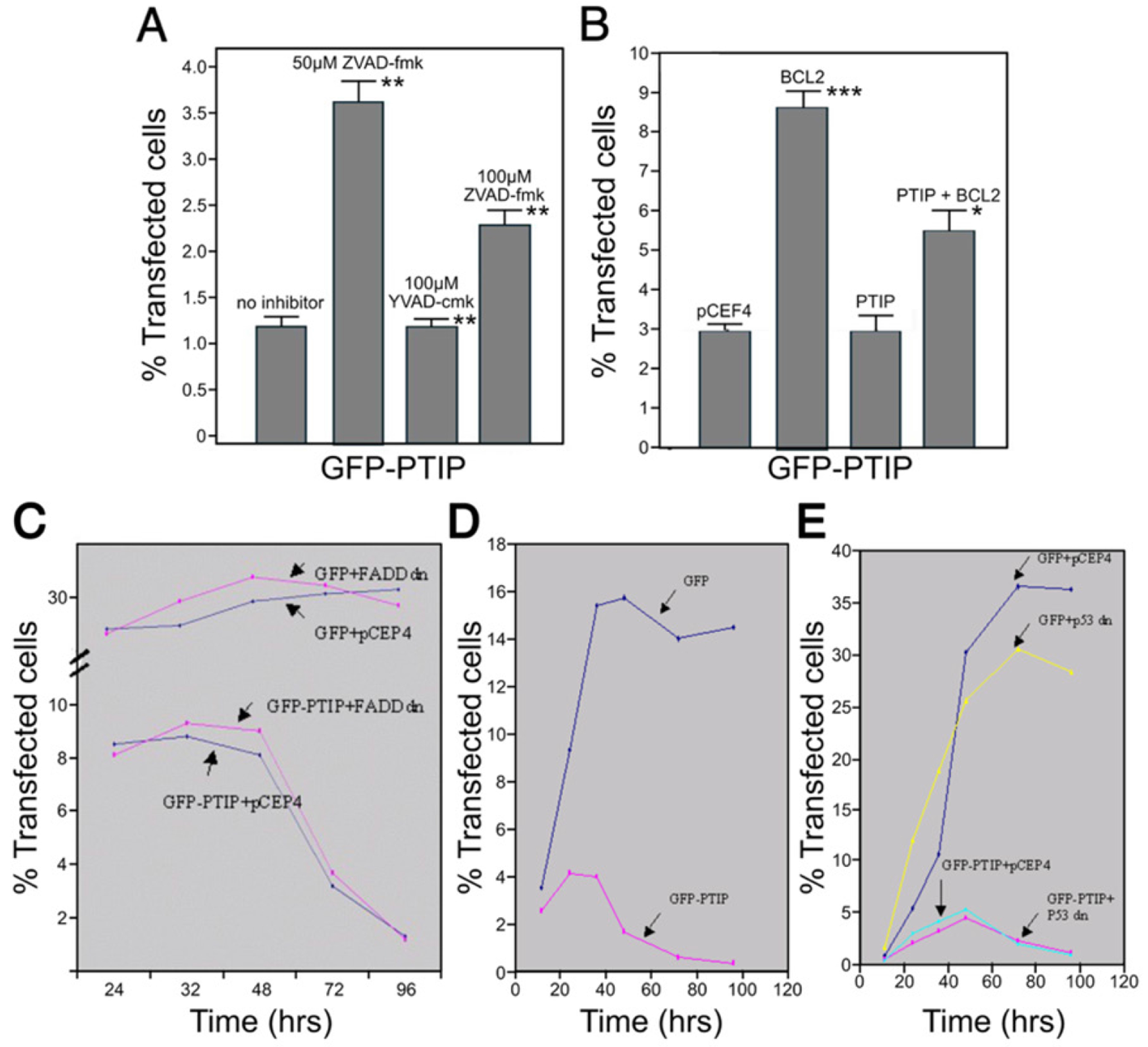
PTIP-induced apoptosis can be inhibited by caspase inhibitors and by expression of Bcl-2 but not by dominant negative FADD nor p53. All experimental values were determined as the average of 3 independent experiments with significance values as p≤0.05*; p≤0.01**; p≤0.001***. (**A**) NIH-3T3 cells were transfected with either pEGFP or pEGFP-PTIP, and 24 hours after transfection, the media was replaced with fresh medium containing caspase inhibitors at concentrations noted in the box. Ninety six hours post-transfection, the percentage of transfected cells were determined by FACS. Blue column, no inhibitors; red column, ZVAD-fmk (50 μM); yellow column, YVAD-cmk (100 μM); green column, DEVD-fmk (100 μM) (each from Clontech). (**B**) Bcl-2 inhibits GFP-PTIP apoptosis. NIH-3T3 cells were transiently transfected with either 0.5 μg pEGFP + 1.5 μg pCEP4-L (control); or 0.3 μg GFP + 1.5 μg pCMV-Bcl-2; or 0.3 μg pEGFP-PTIP + 1.5 μg pCEP4-L; or 0.3 μg pEGFP-PTIP + 1.5 μg pCMV-Bcl-2. 96 after transfection, cells were collected and analyzed by FACS to determine the percentage of transfected cells. (**C**) A dominant negative (dn) form of FADD did not block GFP-PTIP mediated apoptosis. NIH-3T3 cells were transfected with 0.3 μg pEGFP+ 1.5 μg pCEP4; 0.3 μg pEGFP + 1.5 μg pCMV-FADD dn; 0.3 μg pEGFP-PTIP + 1.5 μg pCEP4; 0.3 μg pEGFP-PTIP + 1.5 μg pCMV-FADD dn. The percentage of transfected cells was determined by FACS at the designated time points following transfection. (**D**) GFP-PTIP induced apoptosis in p53 null CHO cells. Transfections and assays were carried out as described in the legend to Figure 4B. (**E**) A dominant negative (dn) form of p53 did not block the apoptotic effect of GFP-PTIP. NIH-3T3 cells expressing dn p53 were transfected with 0.3 μg pEGFP+ 1.5 μg pCEP4; 0.3 μg pEGFP + 1.5 μg pCMV-p53 dn; 0.3 μg pEGFP-PTIP + 1.5 μg pCEP4; 0.3 μg pEGFP-PTIP + 1.5 μg pCMV-p53 dn. The percentage of transfected cells was determined by FACS at the indicated time points following transfection. GFP-PTIP is transported from nucleus to mitochondria and causes mitochondria aggregation. **Abbreviations:** Bcl-2: B-cell leukemia/lymphoma 2 protein; CHO: Chinese Hamster Ovary; DEVD-fmk: Asp-Glu-Val-Asp-fluoromethylketone; FACS: Fluorescence-Activated Cell Sorting; FADD: Fas-Associated Death Domain; GFP: Green Fluorescent Protein; PTIP: PAX-Interacting Protein 1; YVAD-cmk: Acetyl–tyrosyl-valyl-alanyl-aspartyl–chloromethylketone; ZVAD-fmk: Carbobenzoxy-valyl-alanyl-aspartyl-[O-methyl]-fluoromethylketone.

To further confirm the cell death induced by PTIP is apoptotic and to test which caspase is critical, we employed several caspase inhibitors employing the GFP-PTIP assay described above. As shown in Figure 3A, ZVAD-fmk (carbobenzoxy-valyl-alanyl-aspartyl-[O-methyl]-fluoromethylketone), which inhibits a broad spectrum of caspases, showed the strongest blocking activity; ZVAD-fmk, which is specific for caspase-3, showed stronger blocking activity than did YVAD-cmk (acetyl–tyrosyl-valyl-alanyl-aspartyl–chloromethylketone), which is specific for caspase-1. These results indicate that caspases are required for PTIP-induced apoptosis.

Caspase-3, which is mainly activated by the mitochondria pathway and the activation of which can be blocked by overexpression of Bcl-2 protein, appeared to be the more important to follow up. GFP-PTIP was co-transfected with pCMV-Bcl2, which expresses Bcl-2 protein under the control of CMV promoter, into NIH-3T3 cells. After 96 hr, the percentage of transfected cells was analyzed by flow cytometry. As shown in Figure 3B, co-expression of Bcl-2 delayed the rate of cell death by >2-fold relative to control GFP-transfected NIH-3T3 cells. This result implicates Bcl-2 as an inhibitor of PTIP-mediated apoptosis.

### The BRCT3 and 5 domains as well as the polyglutamine-rich (QR) domain of PTIP are critical for apoptosis

BRCT domains are critical for essential functions of numerous BRCT-domain-containing proteins, including PTIP, as reviewed by Pena-Guerrero *et al.* [2]. To determine whether either BRCT3 and/or BRCT5 domains of PTIP are required for its apoptotic activity, we truncated the 5^th^ BRCT domain in pEGFP-PTIP and transfected this mutant (PTIP-BRCT5) into NIH-3T3 cells. As shown in Figure 2A, the deletion of the BRCT5 reduced the apoptotic effect significantly. Thus, the most C-terminal BRCT domain plays an important, but not an essential, role in apoptosis. This suggests that BRCT5 truncation reduced the rate at which apoptotic cells were dying.

We also point-mutated the most conserved threonine (residue 630) in the more N-terminal BRCT3 to methionine. This mutation, termed PTIP BRCTm, has been shown to cause loss-of-function in the BRCT-containing fission yeast cut5/rad4 gene [14]. As shown in Figure 2A, Disruption of Thr630 significantly reduced PTIP-mediated apoptosis.

Finally, we deleted the 181 amino acid QR, termed GFP-PTIP(QR-) and measured its effect as described above by flow cytometry (Figure 2A). Deletion of the polyglutamine-rich domain QR of PTIP significantly decreased apoptosis.

Collectively these data implicate either directly, through mutation of the 3 motifs or indirectly, through the effects on tertiary structure or stability, the importance of the BRCT3 and 5 domains as well as the QR domain. However, more precise point mutations within BRCT5 and QR would refine these conclusions.

### Neither activation of the FAS/CD95 pathway nor p53 is required for PTIP-mediated apoptosis

The FAS/CD95 pathway plays an important role in the differentiation and apoptotic selection of lymphocytes in the immune system. The developmental-stage-specific expression pattern and cytokine inducibility of PTIP mRNA in B cells (Figure 3C) suggested a possible role of FAS/CD95 in its apoptosis effect. A dominant negative (DN) form of FADD (Fas-associated death domain) (pFADDdn), which can bind to FAS but lacks its ability to recruit the downstream procaspase-8, has been shown to block the FAS pathway [15–17]. We co-transfected dominant pFADDdn with GFP-PTIP into NIH-3T3 cells or Hela cells (data not shown) and measured the percentage of transfected cells at different time points by flow cytometry. As shown in Figure 3C, no significant difference in apoptotic induction was observed, suggesting that PTIP does not require an activated FAS pathway to execute its apoptotic function; alternatively, PTIP acts downstream of FADD in this pathway.

We next investigated the potential role of p53 by employing a similar strategy to that above. Two pieces of evidence indicated that p53 had no role in PTIP-mediated apoptosis, First, we observed no significant effect on apoptosis when PTIP was transfected into p53 null CHO cells (Figure 3D). Secondly, a dominant-negative (DN) form of p53, which by tetramerizing with wild type p53 causes its failure to bind DNA [18,19], showed no reduction of apoptosis in NIH-3T3 cells as analyzed by fluorescence-activated cell sorting (FACS) (Figure 3E). Further confirmation was achieved by employing the same assay in COS-7 transfected cells (data not shown).

### PTIP translocation from nucleus to mitochondria results in mitochondrial aggregation during apoptosis

**Figure 4.**
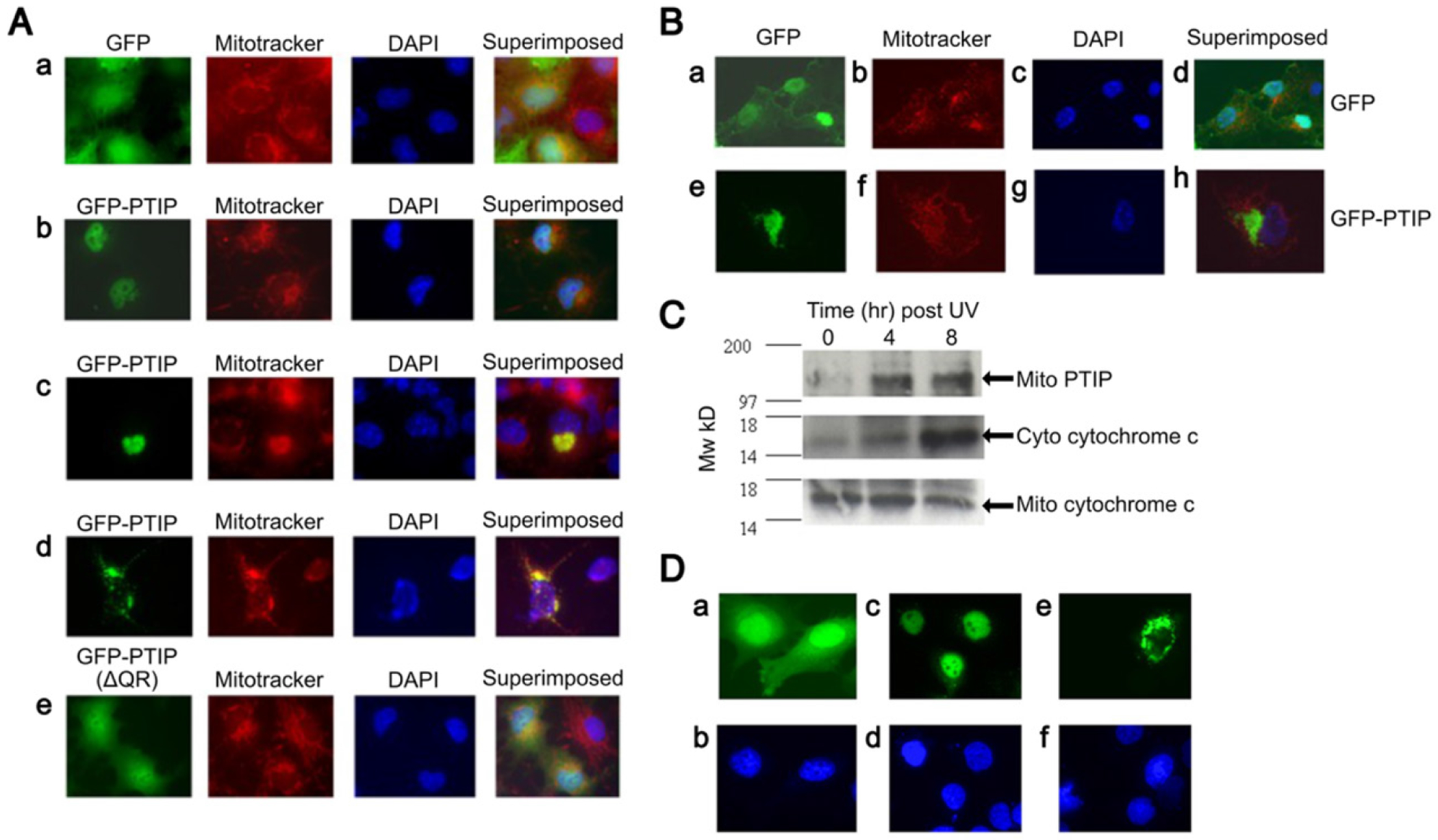
PTIP is transported from nuclei to mitochondria. (**A**) PTIP transport results in mitochondrial aggregation. COS-7 cells were transiently transfected with pEGFP, pEGFP-PTIP or pEGFP-PTIP(QR-) which lacks the polyglutamine-rich 24 hours or 48 hours later, cells were stained with Mitotracker and DAPI prior to observation under fluorescence microscopy. (**a**) GFP control; (**b**) nuclear localization of GFP-PTIP; (**c**) mitochondria localization of GFP-PTIP and mitochondria aggregation; (**d**) another example of mitochondria localization of GFP-PTIP and mitochondria aggregation; (**e**) localization of GFP-PTIP(QR-). (**B**) Translocation of PTIP from nuclei to mitochondria results in cytochrome c release. COS-7 cells were transfected with pEGFP or pEGFP-PTIP. Forty eight hours post-transfection, cells were immunostained with anti-cytochrome c and DAPI and then observed under fluorescence microscopy. (**a–d**) GFP transfected COS-7 cells; (**e–h**), GFP-PTIP-transfected cells; (**b** and **f**), immunostaining with anti-cytochrome c; (**c, g**), DAPI staining; (**d, h**), superimposed. (**C**) Endogenous PTIP is translocated to mitochondria in response to UV-induced apoptosis. Day 0: HeLa cells were seeded at 5 × 10^6^/150 mm dish. Day 1: Cells were treated with UV as previously described in [45]. After further incubation at 0, 4, and 8 hr, cells were harvested, and mitochondrial and cytosolic fractions were prepared as previously described [45]. Aliquots of cytosol protein (10 μg) and mitochondrial protein (5 μg) were fractionated over 8% SDS-PAGE (for detection of PTIP) or over 15% SDS-PAGE (for detection of cytochrome c), transferred to nitrocellulose and probed with anti-cytochrome c or anti-PTIP Abs. The antigen/antibody complexes were visualized by ECL. (**D**) Mutation (m) of the NLS-like sequence of PTIP restricts its localization exclusively to cytoplasm. Hela cells were transiently transfected with 1 μg of pEGFP (**a, b**), or 1 μg of pEGFP-PTIP (**c, d**) or 1 μg of pEGFP-PTIP(NLSm) (**e, f**). Twenty four hr post-transfection, cells were observed via fluorescence microscopy. **b, d** and **f** are DAPI stains of **a, c**, and **e**, respectively. Data shown are representative values of 3 independent experiments. **Abbreviations:** COS-7: CV-1 in Origin with SV40 genes; DAPI: 4’,6-diamidino-2-phenylindole; ECL: Enhanced Chemiluminescence; GFP: Green Fluorescent Protein; NLS: Nuclear Localization Sequences; PTIP: PAX-Interacting Protein 1; QR: Glutamine-rich; SDS-PAGE: Sodium Dodecyl-sulfate Polyacrylamide Gel Electrophoresis.

To characterize the mechanism by which PTIP induces apoptosis, we first identified its subcellular localization. COS-7 cells, transiently transfected with pEGFP-PTIP, were observed via fluorescence microscopy at different time points. At 24 hr post-transfection, while GFP-control transfectants localized evenly between nucleus and cytoplasm (Figure 4A–a), GFP-PTIP are localized within the nuclei of >90% of transfected cells (Figure 4A–b). Yet at 48 hr, >70% of transfected cells are localized within the cytoplasm in a punctate pattern as well as occasionally as large, often perinuclear aggregates near the nuclear membrane (Figure 4A–c, d).

The punctate pattern was reminiscent of that of mitochondria. Thus, we used a mitochondrial dye, Mitotracker, to label them. As shown in Figure 4A–d, aggregated GFP-PTIP co-localized with Mitotracker; a mitochondrial localization was confirmed by anti-cytochrome c oxidase IV immunostaining (data not shown). Similar results were observed in 293 and NIH-3T3 cells, although the percentage of transfected cells with large GFP-PTIP aggregates was lower in NIH-3T3 (data not shown).

These results strongly suggest that PTIP translocated from the nucleus to mitochondria resulting in mitochondrial aggregation. To test whether the QR domain is required for PTIP-induced mitochondrial aggregation, we transiently transfected the QR deletion vector, (pEGFP-PTIP(QR-), into several cell lines. As shown in Figure 4A–e, at 24 hr post-transfection, we observed evenly enhanced expression of this QR-deficient mutant both within the nucleus and the cytoplasm (Figure 4A–e). Even after 48 to 72hr post-transfection, we only observed <1% of the transfectants that expressed PTIP(QR-) in an aggregated pattern.

These results indicated that the QR domain serves a critical function in retaining PTIP within the nucleus and inducing aggregation of mitochondria. We also transiently transfected GFP-PTIP carrying a deletion of the more C-terminal BRCT domain into NIH-3T3 cells or COS-7 cells and found no obvious changes in the localization of PTIP in either cell line (data not shown). Despite this result, and as we described in Figure 4, this mutant had significantly attenuated apoptotic activity, suggesting that mitochondrial aggregation may not be the only mechanism employed by PTIP to induce apoptosis.

### PTIP induces cytochrome c release from mitochondria aggregates

The translocation of PTIP from nucleus to mitochondria, together with the fact that Bcl-2 inhibits PTIP-induced apoptosis, prompted us to investigate the role of mitochondria pathway in this apoptosis process. Moreover, we observed that, at the late stage of transient transfection (72 or 96 hr), a higher percentage of GFP-PTIP in aggregated mitochondria. This suggested that the transfected cells with mitochondrial might be at an early stage of apoptosis. These observations led us to determine if, during this process, cytochrome c is released from mitochondria into cytoplasm.

We employed cytochrome c oxidase immunostaining of the PTIP-transductants described above to determine by fluorescence microscopy whether aggregated PTIP colocalized with cytochrome. We utilized Mitotracker R which stains the inner mitochondrial matrix as a probe. As shown in Figure 4B(e–h), we observed that cytochrome c was released from these aggregated mitochondria in ~90% of transfected cells, whereas GFP-only transfectants retained all cytochrome c in their mitochondria (Figure 4Ba–d). We noted that in some cells with GFP-PTIP aggregates, we observed that cytochrome c still remained in a fraction of non-aggregated mitochondria, whereas in other cells, cytochrome c was released from both unaggregated and aggregated mitochondria. This heterogeneity may reflect the fact that these two cell populations are at different apoptotic stages, i.e., the latter population being at a later stage. This interpretation is supported by the observation that characteristic morphological changes of apoptosis were observed in cells with cytochrome c released from all mitochondria. As anticipated, in control GFP-only transfected cells (Figure 4Aa–d), cytochrome c localized exclusively within mitochondria.

### Endogenous PTIP translocates from nuclei to mitochondria during UV-induced apoptosis

To ensure that the results obtained above in transfected cells were valid, we tested whether endogenous PTIP retained the same phenotype. Hela and NIH-3T3 cells were irradiated with UV light to induce apoptosis.

Briefly, cells were stained with the fluorescent dye, Hoechst 33258, which on binding to DNA, emits blue fluorescence when excited by UV light. Apoptotic cells will exhibit condensed and/or fragmented nuclei. Four and eight hrs following irradiation, we performed subcellular fractionation of nuclei, mitochondria and cytosol and then subjected aliquots of each fraction to Western blot analysis using a commercial polyclonal Ab against PTIP (further detailed in Materials and Methods). As shown in Figure 4C, we observed a significant increase in mitochondrial localization of PTIP at both time points following UV irradiation.

These results indicated that translocation of PTIP from nuclei to mitochondria is not an artifact of GFP-PTIP transduction and is physiologically relevant following normal apoptotic stimuli.

### A bipartite nuclear localization-like sequence restricts PTIP to cytoplasm

Both shown previously [1] and here (Figure 4Dc) PTIP localized primarily (as high as 80%) within the nucleus as compared to GFP-only transfectants (Figure 4D–a). However, evidence presented in this report demonstrated that a significant fraction of PTIP accumulates within the cytoplasm and/or within mitochondria (Figure 4Dc).

We identified within residues 834-847 a region (^834^KRARIEDLPPPTKK^847^) which shared similarity with bonafide bipartite (underlined) nuclear localization sequences (NLS) of p53 and Nucleoplasmin [20,21] and is highly similar to several other shuttling proteins, as reviewed by Liu *et al.* [22]. To test whether this NLS-like sequence plays a role in the nuclear localization of PTIP, we employed site-directly mutagenesis to substitute two typical NLS residues K846E and K847E (denoted above in bold). Then they were cloned into GFP-PTIP, and their localization was analyzed 24 hrs post-transfection into Hela, NIH-3T3 and COS-7 cells.

In contrast with wild type GFP-PTIP, which localized exclusively within the nucleus, the GFP-PTIP double-point mutant (K846E; K847E) localized exclusively within the cytoplasm (Figure 4De). We also noted that the percentage of GFP-PTIP aggregates did not change significantly, suggesting that the failure of nuclear localization did not affect its ability to form cytoplasmic aggregates.

We then tested the effects of the NLS-like mutation on the apoptotic activity of PTIP using the same approach as above but at different time points following transduction. As shown in Figure 4D, the mutation (m) of the NLS-like sequence of PTIP restricts its localization exclusively to cytoplasm. However, the apoptotic activity of this NLS-like mutant, while not totally abrogated, was modestly attenuated (data not shown).

These results suggest that nuclear localization is important but not essential for PTIP apoptotic activity. That PTIP transits first to the nucleus and then to the mitochondria is readdressed in the Discussion section.

### Both the BRCT5 domain and the QR domain are essential for PTIP-induced G_2_/M arrest

Most characterized BRCT domain-containing proteins, including PTIP [13] have been shown to function in DNA replication, DNA repair and/or cell cycle control [2]. The presence of two BRCT domains in PTIP prompted us to investigate its role in cell cycle checkpoint control.

**Figure 5.**
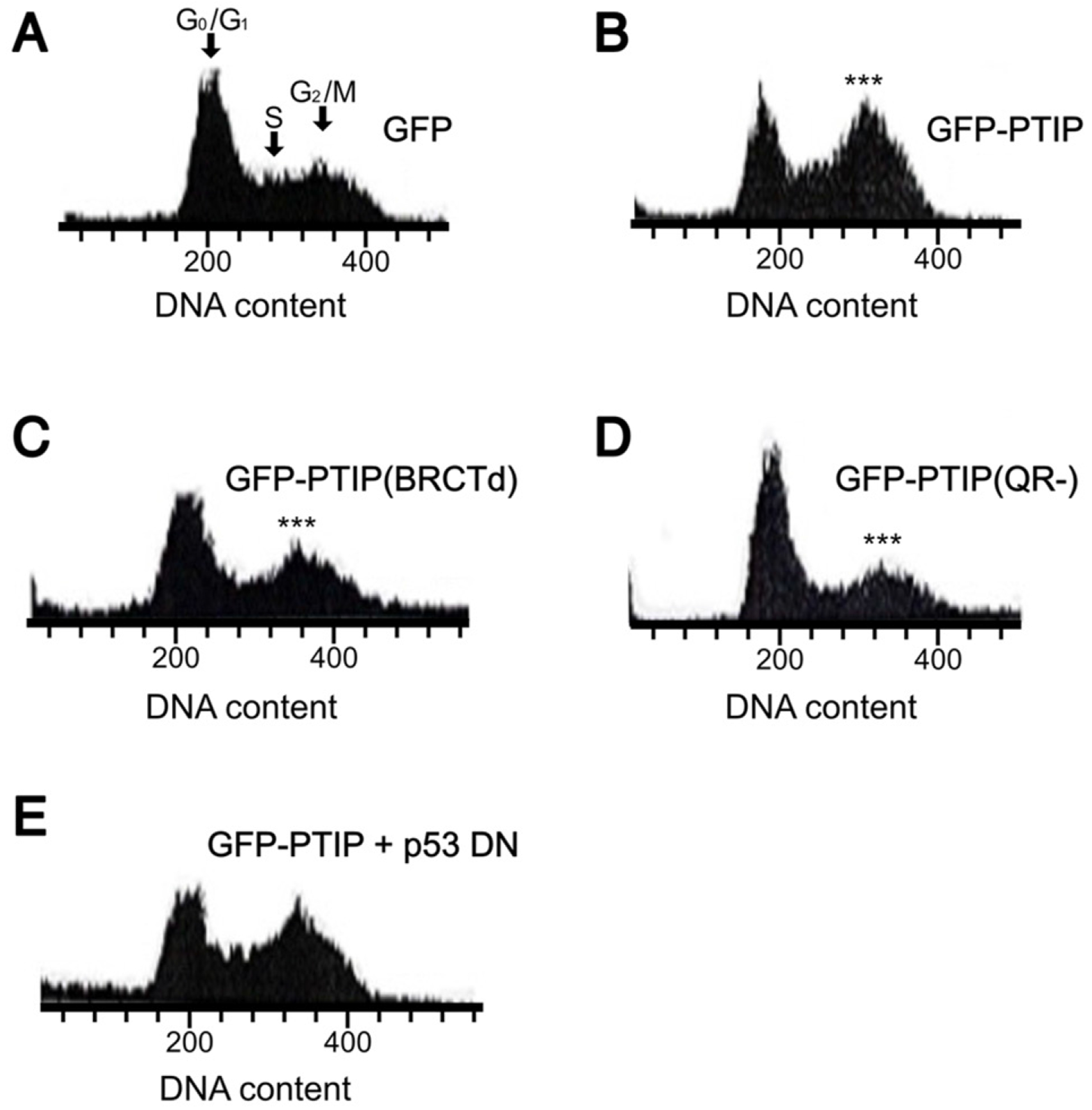
Over-expression of PTIP results in G_2_/M cell cycle arrest. COS-7 cells were transfected with (**A**) ~0.5 μg pEGFP; (**B**) ~1.0 μg pEGFP-PTIP; (**C**) ~1.0 μg pEGFP-PTIP(BRCTd); (**D**) ~1.0 μg of pEGFP-PTIP(QR-); and (**E**) ~0.3 μg pEGFP-PTIP + ~1.5 μg pCMV-p53dn. Forty eight hours post-transfection, cells were harvested and stained with PI. Transfected cells were gated and their DNA content was analyzed by FACS. All experimental values were determined as the average of 3 independent experiments with statistical values of p≤0.05*; p≤0.01**; p≤0.001***. **Abbreviations:** BRCT: BReast Cancer C-Terminal; COS-7: CV-1 in Origin with SV40 genes; DNA: Deoxyribonucleic Acid; FACS: Fluorescence-Activated Cell Sorting; PI: Propidium iodide; PTIP: PAX-Interacting Protein 1.

COS-7 or 293 cells were transiently transfected with pEGFP-PTIP and after 48 hrs were harvested, fixed and stained with PI. Transfected cells were gated, and DNA content was analyzed by flow cytometry. As shown in Figure 5B, the COS-7 transfected cells showed a significantly higher percentage of cells at G_2_/M than GFP-only transfectants (Figure 5A). This indicated that, as anticipated, PTIP over-expression arrests the cell cycle at G_2_/M.

Next, we tested the requirements of the BRCT5 domain as well as the QR domain in this process. As shown in Figures 5C and 5D, either deletion of the BRCT5 (GFP-PTIP(BRCTd) or the QR domains (GFP-PTIP(QR-) significantly decreased PTIP-mediated G_2_/M arrest.

Finally, given the established importance of p53 in G_2_/M arrest [23], we cotransfected a DN form of p53 (p53dn) [23] with pEGFP-PTIP and analyzed the results as above. Unexpectedly, as shown in Figure 5E, blocking p53 expression in COS-7 cells had no significant impact on PTIP’s ability to exert G_2_/M cell cycle arrest.

Collectively These results indicate that several motifs within PTIP contribute to its cell cycle regulation. However, our results are restricted to COS-7 and to 293 cells, and the unexpected lack of a role for p53 must be examined further in additional cells and in more detail.

## Discussion

### PTIP transcription factor-induced apoptosis

We observed that over-expression of PTIP resulted in death of several cell types tested. These included malignant lines transformed at different stages of differentiation as well as in the commonly used mouse fibroblastic line, NIH-3T3. It is important to note that NIH-3T3 cells are not inherently transformed and do not exhibit a cancerous phenotype. While NIH 3T3 are immortalized cells, they undergo malignant transformation only when exposed to oncogenic factors. Also, NIH-3T3 can undergo spontaneous immortalization under specific culture conditions such as high cell density or repeated passaging. Thus, the PTIP mediated apoptotic activity observed in this report is broadly applicable to normal fibroblasts.

PTIP joins a list of a several transcription factors, traditionally known for their roles in gene regulation within the nucleus, to translocate to the mitochondria and directly trigger the intrinsic apoptosis pathway [25–27]. Often this transcription-independent function works in parallel with nuclear activity to amplify the cell death signal. The tumor suppressor protein p53 is the best-studied example of a transcription factor that induces apoptosis from mitochondria. But curiously, we observed that PTIP-mediated apoptosis is independent of p53. However, in both the PTIP and p53 cases, rapid, mitochondrial-driven apoptosis is thought to complement the slower, transcription-dependent activation of pro-apoptotic genes like *Noxa* and *Puma*.

The *c-Myc* oncogene, primarily known for promoting cell proliferation, also induces apoptosis on removal of normal growth and survival signals [25–27]. Its pro-apoptotic activity is often mediated by the mitochondrial pathway. Although the details of *c-Myc*’s mitochondrial translocation mechanism remain mostly unknown, its activation can lead to the release of cytochrome c from the mitochondria. *c-Myc* differs from PTIP and p53 as it appears to indirectly prime the mitochondria for apoptosis by altering the balance of pro- and anti-apoptotic Bcl-2 family proteins.

Three other well studied transcription factors, ATF6 (Activating Transcription Factor 6), STAT3 (Signal Transducer and Activator of Transcription 3) and TR-3, employ mitochondria but for different purposes. ATF6 is activated by endoplasmic reticulum (ER) stress and induces apoptosis by promoting the transcription of pro-apoptotic genes that target mitochondria. STAT3 modulates calcium flux between the ER and mitochondria, and if deregulated, it can lead to mitochondrial calcium overload and trigger apoptosis. The TR-3 orphan receptor is another nuclear factor that was shown, in response to apoptotic stimuli, to translocate from the nucleus to mitochondria and cause apoptosis by induction of cytochrome c release [25–27].

However, there is no evidence that any of the above transcription factors caused mitochondria aggregation—a mechanism associated with PTIP-mediated apoptosis (discussed below).

### The roles of caspases and Fas ligand in PTIP-triggered apoptosis

Our data showed that PTIP-induced apoptosis is most strongly activated by caspase-3 and Fas/CD95. Caspase-3 is activated by Fas/CD95 via the caspase-8/ mitochondria pathway. This was consistent with our finding that over-expression of Bcl-2, which typically inhibits the apoptosis activated through mitochondria pathway but not necessarily apoptosis induced by Fas/CD95, significantly weakened the apoptotic effect of PTIP. Thus, the Bcl-2 inhibition observed here may depend on its ability to block caspase-9 activation by blocking cytochrome c release (discussed further below). Although caspase-3 plays a critical role for PTIP apoptotic activity, our data revealed that it is not the only caspase involved, as the strongest inhibitory activity was via the broad-spectrum caspase inhibitor ZVAD-fmk. Thus, additional caspases other than caspase-3 are downstream of PTIP.

We further observed that blockage of the FAS/CD95 pathway by a dominant negative form of FADD had no significant effect on PTIP’s apoptotic activity. Dominant negative FADD acts by blocking the recruitment and activation of caspase-8. Thus, caspase-8 activation via FAS trimerization is not required. It can be further suggested that an activated Fas/CD95 pathway is not required for the apoptosis induced by PTIP and/or that PTIP acts downstream of caspase-8 recruitment.

In response to DNA damage induced by a variety of stimuli, including γ-irradiation, accumulation of the p53 protein results in cell cycle arrest or apoptosis, depending on the degree of genomic damage. Our data indicated that PTIP-induced apoptosis is independent of p53 as supported by two lines of evidence: (1) blocking of the p53 pathway by a DN form of p53 had no significant effect on PTIP’s apoptotic activity in NIH-3T3 cells. (2) PTIP-induced apoptosis was inhibited in in CHO cells carrying a p53 null mutation. However, these results do not exclude the possibility that PTIP acts downstream of the p53 pathway.

### BRCT domains are essential for PTIP-induced apoptosis

Our data revealed that two BRCT domains are important for PTIP’s apoptotic activity. Both deletion of the more C-terminal BRCT or the point mutation of the most conserved threonine within the more N-terminal BRCT significantly decreased PTIP-induced apoptosis. BRCT domains are essential to the functions of many genes. Crystallographic and biochemical evidence revealed that BRCT-containing proteins act as protein-protein interaction domains by providing a scaffolding role to recruit other proteins to form complexes (reviewed in [24]). It is conceivable that PTIP may act as a scaffold to recruit other required apoptotic factors. We tested the *in vitro* interactions between PTIP BRCTs and several other BRCT-containing proteins (e.g., XRCC1, PARP, DNA ligase IIIα and Ect2) but observed no specific interactions (data not shown). These data, along with the majority of BRCT interactions previously identified, required components of DNA repair complexes. Thus, our negative data suggests that PTIP-mediated apoptosis is not mediated via DNA repair complexes, but clearly, more work in identifying PTIP complexes is required.

Alternatively, one or more of PTIP’s BRCT domains might act as transcriptional activator motifs when fused to a DNA binding domain. It was previously shown that PTIP associated with RNAP polymerase II holoenzyme via complexes with PAX2 and PAX5 [1,7,8]. It is reasonable that one of the mechanisms by which PTIP induces apoptosis is through potential transactivation activity of its BRCT domains. If this is the case, it will be of interest to further identify the transcription targets of PTIP.

Although our data suggests that the mitochondria pathway plays a major role in PTIP-induced apoptosis, several lines of evidence suggest other pathways may also be involved in this process. First, in PTIP knockout mice, the level of cdc2 kinase (Cyclin-dependent kinase-1[CDK1]) is significantly reduced as compared with wild type mice [1]. This indicated that PTIP can up-regulate the level of cdc2 by, as yet, an unidentified mechanism. Others have shown that up-regulation of CDK levels is critical in several apoptotic events [28]. It would be interesting to determine whether cdc2 levels contribute to PTIP-induced apoptosis.

Also, protein aggregation can impair the ubiquitin-proteosome system, which subsequently leads to cell cycle arrest and apoptosis [29]. As discussed below, and quite evident in the data of Figure 4, mitochondrial aggregation is observed during apoptosis induced by PTIP. It is conceivable that impairment of ubiquitin-proteosome system by this aggregation may be another mechanism involved.

### NLS-like sequences contribute to PTIP-mediated apoptosis

Point mutations K846/E and K847/E within the region of PTIP which resembled the NLS of p53 and Nucleoplasmin [20,21] suggested that PTIP needs to transit to the nucleus prior to and as a requirement to mobilize within mitochondria. This interpretation is consistent with the observation that the above mutations showed no detectable defect in the ability of PTIP to promote mitochondrial aggregation. Perhaps, the induction of mitochondrial aggregation is not the only mechanism by which PTIP induces apoptosis? That hypothesis is consistent with our observation that BRCT3 truncation still allowed aggregate formation but significantly attenuated PTIP-mediated apoptosis. The involvement of mechanism(s) other than via the mitochondria pathway also is consistent with our observation that expression of Bcl-2 and caspase inhibitors only partially inhibited PTIP-mediated apoptosis.

### The role of polyglutamine repeats (QR) in PTIP-mediated aggregation and apoptosis

Among the age-dependent protein aggregation disorders, nine neurodegenerative diseases are caused by expansions of cytosine-adenine-guanine (CAG)-triplet repeats encoding polyglutamine (polyQ) tracts (reviewed in [30]). These aggregates may contain additional proteins, including ubiquitin and ubiquitin-binding proteins, proteasome components, chaperones, and in some cases, transcription cofactors. PolyQ disorders in the expression of mitochondrial proteins was first observed in Huntington’s Disease (HD). This was not unsuspected given the subacute, systemic inhibition of oxidative phosphorylation within the striatal neuropathological changes of HD [31,32].

Here we observed that deletion of the QR domain in PTIP led to the failure of protein aggregation in mitochondria—directly implicating this glutamine-rich region as critical for protein aggregation in mitochondria. This failure of PTIP to aggregate within mitochondria also led to the failure of mitochondria to release cytochrome c thereby weaking the apoptotic effect of PTIP.

These results demonstrate that, as in HD, the PTIP-bearing QR domain can induce protein aggregation in organelles other than the nucleus and contribute to apoptosis by mechanisms other than causing nuclear inclusion. We also noted brighter expression of GFP-PTIP lacking the QR domain in transfected cells, suggesting that the deleted form of the fusion protein was more stable than wild type GFP-PTIP.

### Overexpression of PTIP results in G_2_/M cell cycle arrest

In Figure 5 we demonstrated that over-expression of PTIP caused G_2_/M cell cycle arrest. This observation corroborated data previously observed for PTIP [1,33,34] as well as for several other BRCT domain-containing proteins (reviewed in [35]). Our observations are consistent with data from the Dressler laboratory showing that cultured blastocysts from late embryos of PTIP knockout mice die from chromosomal fragmentation because of the failure to execute the G_2_/M checkpoint [33]. We extended those observations by demonstrating that deletion ofthe glutamine-rich QR domain of PTIP abrogates G_2_/M arrest. Given that truncation of the QR domain resulted in failure of PTIP-mediated aggregation, it can be inferred that PTIP aggregation is required for G_2_/M arrest. This hypothesis is consistent with previous observations that protein aggregation can, in itself, induce G_2_/M arrest as a protective mechanism. This may allow the cell to pause before entering mitosis so that repair mechanisms can address the damage caused by aggregated proteins (reviewed in [36]). Of future interest is to determine whether the observed G_2_/M arrest is, at least partially, dependent upon PTIP-induced mitochondrial aggregation.

The BRCT domains also play important roles in this process: The truncation of the BRCT5 domain abrogated PTIP-induced G_2_/M arrest. In its role in apoptosis, PTIP also may function as a scaffold to recruit other factors required for G_2_/M arrest through their interactions with either BRCT3 or 5 domains. Based on our observation that truncation of BRCT5 only weakened the ability of PTIP to promote aggregation, we can conclude that aggregation alone is insufficient to cause G_2_/M arrest.

We cannot rule out the possibility that other mechanisms may exist. For example, in PTIP knockout mice, the level of p27^kip1^ is significantly reduced, suggesting that over-expression of PTIP may up-regulate p27 sufficiently to result in G_2_/M arrest. This is consistent with the finding that PTIP can physically interact with transcription factors Pax2 and Pax5 [1]. These observations raise the possibility that PTIP may function as a transcription factor or co-factor to regulate p27. However, it seems contradictory that PTIP would up-regulates both cdc2 (as discussed above) and p27, as these two factors basically perform opposite roles in the cell cycle. But during development or under physiological conditions, up-regulation of cdc2 and p27 may occur within different time windows to avoid this conflict.

As shown previously [37], the phenotype of PTIP^−/−^ cells is similar to that of ATR^−/−^, ATM^−/−^ and BRCA1^−/−^ phenotypes, including abnormality in G_2_/M checkpoint control and sensitivity to treatment with DNA damaging agents. Each of these proteins can function in the same pathway by regulating G_2_/M cell cycle arrest and other aspects of cell growth. These include both its BRCT domains as well as its QR domain.

### PTIP is targeted to mitochondria in the absence of a canonical targeting sequence

That PTIP-mediated apoptosis functions via the mitochondrial pathway raised the question as to what targets it there from the nucleus. A canonical mitochondrial targeting sequence (MTS) typically contains at its N-terminus a relatively short stretch (15–40 amino acids) that bear a high density of positively charged basic residues critical for directing the transport [38]. It is characterized by a positively charged amphipathic helix structure which is recognized by outer or inner mitochondrial membrane (translocases) [38].

PTIP lacks all of these characteristics. As shown in Figure 1, the first two of PTIP’s 6 BRCA domains occupy the N-terminus (residues 13–175) with no significant runs of basic residues nor amphipathic helices. Our analyses of Figure 4, which employed a standard matrix stain (Mitotracker Red), indicated that PTIP is targeted exclusively to the mitochondrial matrix (MPP) but to neither the inner or outer mitochondrial membrane. While examples of C-terminal MTSs are rare, targeting is typically observed to the outer mitochondrial membrane rather than translocation within the matrix [38].

Perhaps the lack of a prototypic MTS should not be anticipated as Schuldiner’ group [39] recently estimated that no more than 25% of mitochondrial proteins use canonical MTS targeting sequences. Plus, several studies have shown that mitochondrial transfer often requires carrier proteins. For example, PINK1 (PTEN Induced Kinase 1) is required to transport the E3 ligase PARKIN into the outer mitochondrial zone [40].

### Large PTIP cytoplasmic aggregates are essential to PTIP-mediated apoptosis

In addition to the perinuclear aggregates of PTIP, we observed numerous PTIP-containing puncta within the cytoplasm. Although their punctate pattern looked similar to that of mitochondria, PTIP typically did not colocalize with Mitotracker (data not shown). This indicated that this fraction of PTIP remained in the cytoplasm outside of mitochondria. However, we observed in some cells that cytochrome c was released from all mitochondria, suggesting that these cells may be at a later stage of apoptosis. This observation is supported by our finding that deletion of the C-terminal QR domain of PTIP significantly reduced aggregation, leading to an evenly distribution within the nucleus and the cytoplasm. This failure in its translocation to and its aggregation within mitochondria significantly weakened the apoptotic effect of PTIP.

To test whether our observations of PTIP puncta were biased by employing transfected PTIP, we tested translocation of endogenous PTIP from the nucleus to mitochondria during UV-induced apoptosis. That we observed quite similar results suggest that our GFP-fusion interpretations are not an artifact and are physiologically relevant apoptosis.

### PTIP aggregates resemble biomolecular condensates created by phase separation

Following translocation from the nucleus, cytoplasmic PTIP localized in a punctate pattern which often resembled perinuclear aggregates (Figure 4). In addition, and while quantitative image analyses were not performed, the fluorometric intensities of these aggregates appeared to reach micron- or submicron-sized. These structures are reminiscent of those observed in liquid-liquid phase separation (LLPS)—a process that produces droplet-like structures termed biomolecular condensates (recently reviewed by Torun *et al.* [41]. Unlike classical organelles, condensates lack membranes and function by compartmentalizing large complexes of homo- or heterogenous molecular aggregates within distinct regions. This process provides cells another mechanism whereby processes as distinct as macromolecular assembly or gene regulation can be controlled. Relevant examples (reviewed by Cao *et al.*, [42]) of condensation include the activation of a number of signaling pathways (eg, B cell receptor [BCR], Retinoic acid-inducible gene I [RIG-1], Nuclear factor kappa B [NF-κB] and T cell receptor [TCR]) and, particularly pertinent to our case, apoptosis.

Prompt clearance of apoptotic cells, a requirement for preventing inflammatory responses, is disrupted by caspases. We identified two of those, caspases 1 and 7, as critical to the mitochondrial-based mechanism of PTIP (Figure 3). These and other caspases often exist in a ribonucleoprotein (RNP)-condensate which are formed reversibly under stress to inhibit further caspase activities. This ultimately prevents excessive apoptosis by eliminating harmful substances via autophagy—an essential quality control of caspase—mediated apoptosis [43].

Future experiments are required to determine if condensation is employed as a critical component of PTIP-mediated apoptosis. One possibility is that PTIP serves as a “scaffold”, as it does for RNAPII [1,7,8], to partition “client” molecules via networks through multiple folded domains. Examination of the high resolution PTIP crystal structure [44] revealed a compact, beta-barrel fold with five antiparallel beta-strands. Included in the beta-barrel is an SRC homology 3 (SH3) domain, which often plays a crucial role in protein-protein interactions. This motif would be a reasonable target for mutation.

A second type of weak multivalent interaction that has been shown to mediate condensation is characterized by intrinsically disordered regions (IDRs). While the native structure of PTIP lacks IDRs, we recently cloned and are characterizing a previously undescribed, shorter isoform of PTIP, which by virtue of frameshift and alternative pre-mRNA splicing encodes a unique C-terminal 50 amino acid (Huang *et al.*, submitted). We plan further studies of both PTIP isoforms to genetically manipulate the multiple PTIP valencies observed in Figure 4. This will allow us to test the hypothesis of phase separation and to define what specific domains might be driving LLPS.

## Materials and Methods

### Plasmids and DNA manipulations

Plasmid pEGFP-C1 was obtained from Clontech. Plasmid pEGFP-PTIP was constructed as described in the follow procedures: Oligonucleotides PTIP-U (5’-GACCTGCTCGAGGATCCCCGGGAATTC-3’) and oligonucleotides PTIP (5’-GGACTAGTTGTGGGCTCTTAGGC-3’) were used as primers and pMYC-PTIP (Lechner *et al.*, 2000) as template to do polymerase chain reaction (PCR) to amplify the full length PTIP cDNA. The PCR product was then cloned into pCR3.1 (Eukaryotic TA Cloning kit, Invitrogen). PTIP was released from pCR3.1 by Xho I digestion and subcloned into Xho I-disgested pEGFP-C1 to make pEGFP-PTIP. To truncate the QR domain, pEGFP-PTIP was digested with Ava I, and the two larger bands were purified from agarose gel. The purified DNA fragments were then ligated to make pEGFP-PTIP(QR-). The deletion of the 5^th^ C-terminal BRCT domain (from amino acid 855 to amino acid 934) was done as described in the following procedures: two pairs of oligonucleotides (Pvu1-U:5’-CAGAGCTGACGCCTCGATCGTTAGTGCTCTTCAC-3’;Pvu1-L:5’-CTGAAGAGCACTAACCTAACGATCGAGGCGTCAGCTCTG-3’;Pvu2-U:5’-CACATTGTGACTCCGGGAG-3’;Pvu2-L:5’-CTCCTCCAGCCAGTCCGATCGCGGAGTCACAATGTG-3’) were used to introduce two PvuI sites flanking the most C-terminal BRCT domain 5 into pEGFP-PTIP by QuikChange site-directed mutagenesis kit (Stratagene). The BRCT domain was then deleted by digestion with Pvu I. The conserved Thr^630^ in the third BRCT domain of PTIP was mutated to Methionine (M) using QuikChange site-directed mutagenesis kit (Stratagene). Oligonucleotides TM-U (5’-CACAAGCCGATGTATGCACCTCCTCTGCGC-3’) and oligonucleotides TM-L (5’-GCGCAGAGGAGGTGCATACATCGGCTTGTG-3’) were used as primers and pEGFP-PTIP as template to do the site-directed mutagenesis. All constructs were confirmed by nucleotide sequencing. To mutate the NLS-like sequence in PTIP, we employed oligonucleotides NLS (K846, 847N)-U: 5’-CCACCTCCCACTAACAACCTGACTCCAG and nucleotides 5’-CTGGAGTCAGGTTGTTAGTGGGAGGTGG-3’ as primers and pEGFP-PTIP as template to replace Lys^846^ and Lys^847^ with Asp using a QuikChange site-directed mutagenesis kit (Stratagene). All constructs were confirmed by nucleotide sequencing. The plasmid encoding dominant negative p53 (pCMV-p53dn) was obtained from Dr. David Johnson (Science Park Research Division, MD Anderson Cancer Center of the University of Texas). The dominant negative FADD expression vector (pCMV-FADDdn) was obtained from Dr. Joseph Nevins (Depart. of Mol. Genetics and Microbiology; Duke Institute for Genome Sciences Duke University).

### Cell culture and transfections

NIH-3T3, Hela, COS-7, 293 and CHO cells were all grown in Dulbecco’s modified Eagle’s medium (DMEM) containing 100 U/ml of penicillin and 100 μg/ml of streptomycin sulfate supplemented with 10% fetal calf serum (GIBCO, Grand Island, N.Y.). For transient transfection, 2 X 10^5^ cells were seeded in a well on a 6-well plate in DMEM medium and grown in monolayer at 37°C in an atmosphere of 5% CO_2_. Eighteen hours later, the cells were transfected using FuGENE 6 transfection reagent (Roche). For pEGFP-PTIP and different truncation and mutation constructs, 1.0 μg of DNA was used for each well; 0.5 μg of pEGFP was used for each well as control. For GFP-PTIP and Bcl-2 co-transfection, 0.3 μg of pEGFP-PTIP and 1.5 μg of Bcl-2 were used for each well; 0.3 μg of pEGFP-PTIP and 1.5 μg of pCEP4 were used as control; 0.3 μg pEGFP plus 1.5 μg Bcl-2 and 0.3 μg pEGFP plus 1.5 μg pCEP4 were also used as control. As described above, COS-7 and 293 cells were seeded and transfected in each well with ~0.5 μg of pEGFP and ~1.0 μg of pEGFP-PTIP and its truncation. For co-transfection, ~0.3 μg of pEGFP-PTIP and ~1.5 μg of pCMV-p53dn were used for each well. Approximately 0.3 μg of pEGFP-PTIP + ~1.5 μg of pCEP4 (mammalian expression vector), ~0.3 μg of pEGFP + ~1.5 μg of pCMV-p53dn and ~0.3 μg of pEGFP-PTIP + ~1.5 μg of pCEP4 were used for each well as control.

For each transfection, we titrated several concentrations of PTIP to optimize and/or normalize its expression within a particular experiment ranging typically from 1.5 μg down to 0.25 μg.

### Flow cytometry

Cells were seeded and transfected as described above. At different time points after transfection, cells were washed by 2 ml phosphate-buffered saline (PBS) and trypsinized for 5 min. The cells were then washed off the plate by 5 ml of DMEM medium and the cell suspension was transferred to a 15 ml culture tube. The cells were collected by centrifugation at 2,000 rpm for 10 min and the cell pellets were washed by PBS once and re-centrifuged at 2,000 rpm for 10 min. The cell pellets were resuspended in 0.5 ml of PBS for flow cytometry analysis (BD FACSCalibur Flow Cytometry System).

### Annexin V staining

CHO cells were plated and transfected as described above. Seventy two hours after transfection, cells were collected and washed twice with cold PBS and resuspended in 1X Binding Buffer (10 mM HEPES [4-(2-hydroxyethyl)-1-piperazineethanesulfonic acid], PH 7.4; 140 mM NaCl; 2.5 mM CaCl_2_) at a concentration of ~1X10^6^ cells/ml. 100 μl of the resuspended cells (~1X10^5^ cells) were then transferred to a 5 ml culture tube and 5 μl of Annexin V-PE (BD Pharmingen) was added. The cells were gently mixed and incubated for 15 min at room temperature in the dark. Four hundred microliters of 1X Binding Buffer was added to each tube and the samples were analyzed by flow cytometry (BD FACSCalibur Flow Cytometry System) as soon as possible (within 1 hours).

### Sub diploid DNA content assays

Cells were transfected with 0.5 μg of pEGFP or pEGFP-PTIP. Seventy two hours later, cells were trypsinized and collected by centrifugation at 2,000 rpm for 5 min. The cells pellets were then resuspended in 300 μl of PBS and 5 ml of ice-cold 75% ethanol was added dropwise under agitation to the cell suspension and stored at −20°C until further use. The cells were then collected by centrifugation at 2,000 rpm and the cell pellets were washed with PBS and then resuspended in PBS containing 50 μg/ml propidium iodide (Sigma) and 50 μg/ml DNase-free RNAase A (Boehringer Mannheim). The cell suspension was incubated for 15 min on ice and protected against light and then analyzed with a Flow Cytometer (BD FACSCalibur Flow Cytometry System).

### Caspase inhibitor assays

NIH-3T3 cells were plated and transfected as described above. Twenty four hours after transfection, culture medium was removed and replaced with new medium with or without caspase inhibitor (the final concentration of ZVAD-fmk: 50 μM; YVAD-cmk: 100 μM; DEVD-fmk (Asp-Glu-Val-Asp-fluoromethyl ketone): 100 μM). Ninety six hours after transfection, percentage of transfected cells was determined by flow cytometry. The data shown were obtained from three independent experiments.

### Transfection and Mitotracker staining

Approximately 2 × 10^5^ COS-7 cells were seeded in 6-well plate in DMEM medium (Dulbecco’s modified Eagle’s medium containing 100 U/ml of penicillin and 100 μg/ml of streptomycin sulfate supplemented with 10% fetal calf serum) and grown in monolayer at 37°C in an atmosphere of 5% CO_2_. Approximately 18 hrs later, the cells were transfected with 1 μg of pEGFP-PTIP or 1 μg of pEGFP-PTIP or with the substitution and truncation mutants described above using FuGENE 6 transfection reagent (Roche). Approximately 24 hrs after transfection, DMEM medium was replaced with fresh DMEM medium with 50 ng/ml Mitotracker (Molecular Probes) preincubated at 37°C. Cells were continued to incubate at 37°C for 30 min and washed with PBS 3X. Cells were then fixed in freshly prepared 3% paraformaldehyde in PBS at room temperature for 30 min. The fixed cells were washed 3X in PBS for 5 min each and then stained with 1 μg /ml DAPI (4′,6-diamidino-2-phenylindole) in Buffer D (10 mM) PIPES [piperazine-N,N′-bis(2-ethanesulfonic acid], 0.1% Triton X-100, 2 mM MgCl_2_, 0.1M NaCl) for 5 min. Then cells were washed in PBS for 5 min and examined boy fluorescence microscopy.

### Immunofluorescence

Adhesive COS-7 cells were seeded at ~1x10^4^ cells/chamber slide (Nalge Nunc International) in DMEM medium supplemented with 10% (v/v) fetal calf serum, and then they were grown in monolayers at 37°C in an atmosphere of 5% CO_2_. Approximately 18 hours later, COS-7 cells were transfected with 0.25 μg of pEGFP-PTIP or 0.25 μg of pEGFP using FuGENE 6 transfection reagent (Roche). About 48 hr later, the medium was removed, and the cells were washed three times in PBS followed by fixation in freshly prepared 3% paraformaldehyde in PBS for 10 min. The fixed cells were washed 3X each in PBS for 15 min followed by permeabilization in 0.15% Triton X-100 in PBS for 15 min. The cells were then blocked for 60 min in blocking buffer (2% bovine serum albumin in PBS) followed by four-hour incubation with a mouse monoclonal antibody against cytochrome c (Pharmingen 6H2, 1:200 dilution). The cells were washed three times at 10 min each in blocking buffer followed by one-hour incubation with Texas-red conjugated goat anti-mouse antibody (1:500 dilution) (Molecular Probe). The immunostained cells were washed three times at 10 min each in PBS followed by staining with 1 mg/ml DAPI and examined via fluorescence microscopy.

### UV-induction of apoptosis, subcellular fractionation and Western blot analyses

On day 0, Hela cells were set up at ~5 × 10^6^/150 mm dish. On day 1, cells were treated with UV as previously described [27]. After further incubation for the indicated times (Figure 4C: 0, 4 and 8 hrs) cells were harvested and mitochondrial and cytosolic fractions were prepared as previously described [45]. Briefly, the irradiated cells were washed once with 1 x phosphate-buffered saline (PBS) and harvested by centrifugation at 800 g for 5 min at 4°C. The cell pellets were resuspended in 3 volumes of Buffer A (20 mM Hepes-KOH, pH 7.5, 10 mM KCl, 1.5 mM MgCl_2_, 1 mM sodium EDTA, 1 mM sodium EGTA [thylene glycol-bis(β-aminoethyl ether)-N,N,N′,N′-tetraacetic acid], 1 mM DTT [dithiothreitol], 250 mM sucrose, and 0.1 mM PMSF [phenylmethylsulfonyl fluoride]) for 15 min on ice followed by passing 15 times through 23 G needle using a 1 ml syringe. The nuclear pellet and unbroken cells were pelleted by centrifugation 1,000 g for 15 min at 4°C. The resulting supernatants were further centrifuged at 10,000 g for 30 min at 4°C to collect mitochondria. The resulting supernatants were further centrifuged at 100,000 g for 30 min at 4°C. The final, remaining supernatants were employed as cytosol controls. Aliquots of ~10 μg of protein from the cytosol and ~5 μg of protein from the mitochondria were subjected to 8% SDS-PAGE (sodium dodecyl-sulfate polyacrylamide gel electrophoresis) (for detection of PTIP) or to 15% SDS-PAGE electrophoresis (for detection of cytochrome c). Following transfer to nitrocellulose, the filters were probed with mouse monoclonal antibody against cytochrome c (Pharmingen 7H8, 1:500 dilution) or with chicken polyclonal antibody against PTIP (1:4000 dilution). The antigen/antibody complexes were visualized by ECL (Enhanced chemiluminescence).

### Cell cycle assays

COS-7 cells were transfected with ~0.5 μg pEGFP or with ~1.0 μg pEGFP-PTIP or ~1.0 μg pEGFP-PTIP(BRCTd) or with ~1.0 μg of pEGFP-PTIP(QR-) or with ~0.3 μg pEGFP-PTIP + ~1.5 μg pCMV-p53dn. Approximately 8 hrs following transfection, cells were trypsinized and collected by centrifugation at 2,000 rpm for 5 min. The cell pellets were then resuspended in 300 μl of PBS, and then 5 ml of ice-cold 75% ethanol was added dropwise under gentle agitation to the cell suspension. The suspensions were stored at −20°C until further use. The cells were then collected by centrifugation at 2,000 rpm and the cell pellets were washed with PBS and then resuspended in PBS containing 50 μg /ml PI (Sigma) and 50 μg/ml DNase-free RNAase A (Boehringer Mannheim). The cell suspension was incubated in the dark for 15 min on ice and then analyzed by flow cytometry (BD FACSCalibur Flow Cytometry System).

### Statistical analyses

Students T-test was used for all statistical comparisons (n=3 independent observations).
